# Esterification of Docosahexaenoic Acid Enhances Its Transport to the Brain and Its Potential Therapeutic Use in Brain Diseases

**DOI:** 10.3390/nu14214550

**Published:** 2022-10-28

**Authors:** Amanda Lo Van, Nathalie Bernoud-Hubac, Michel Lagarde

**Affiliations:** Univ Lyon, INSA Lyon, CNRS, LaMCoS, UMR5259, 69621 Villeurbanne, France

**Keywords:** docosahexaenoic acid, phospholipids, lysophospholipids, blood-brain-barrier, neuroprotection, neurological diseases

## Abstract

Docosahexaenoic acid-containing lysophosphatidylcholine (DHA-LysoPC) is presented as the main transporter of DHA from blood plasma to the brain. This is related to the major facilitator superfamily domain-containing protein 2A (Mfsd2a) symporter expression in the blood–brain barrier that recognizes the various lyso-phospholipids that have choline in their polar head. In order to stabilize the DHA moiety at the *sn*-2 position of LysoPC, the *sn*-1 position was esterified by the shortest acetyl chain, creating the structural phospholipid 1-acetyl,2-docosahexaenoyl-glycerophosphocholine (AceDoPC). This small structure modification allows the maintaining of the preferential brain uptake of DHA over non-esterified DHA. Additional properties were found for AceDoPC, such as antioxidant properties, especially due to the aspirin-like acetyl moiety, as well as the capacity to generate acetylcholine in response to the phospholipase D cleavage of the polar head. Esterification of DHA within DHA-LysoPC or AceDoPC could elicit more potent neuroprotective effects against neurological diseases.

## 1. Introduction

Lipids are major constituents of living cells, as they are important structural components of cell membranes. Polyunsaturated fatty acids (PUFAs) are long-chain fatty acids (18 carbons or more) that contain two or more double bonds. Depending on the location of the last double bond, PUFAs are classified into families such as omega-3 (last double bond on the third carbon starting from the methyl group) and omega-6 (last double bond on the sixth carbon from the methyl group). Contrary to fatty acids that can be synthesized in the human body, some of them cannot be produced de novo and must be incorporated through diet [1]. The latter are called essential fatty acids, and they include omega-6 family precursor linoleic acid (18:2*n*-6) and omega-3 family precursor α-linolenic acid (LNA, 18:3*n*-3). By a cascade of alternating desaturase and elongase enzymatic reactions, which are common to both families, longer PUFAs are biosynthesized from their respective precursors [2]. PUFAs are mainly found esterified within glycerophospholipids present in cell membranes at the *sn*-1 and *sn*-2 positions. Glycerophospholipids are grouped by the structure of their polar head group on the *sn*-3 position. Their amphiphilic nature (one hydrophilic head group and two hydrophobic fatty acids) confers fluidity and selective permeability to the membranes they constitute [3,4]. They are also precursors for signaling metabolites, including eicosanoids, growth hormones, and regulators, and they participate in important physiological processes, such as anti-inflammatory or pro-inflammatory responses [5,6,7,8].

There is a specific enrichment of essential fatty acids in human tissues, notably in the retina, brain, and heart. Contrary to arachidonic acid (ArA, 20:4*n*-6), which is the major PUFA of human tissues, docosahexaenoic acid (DHA, 22:6*n*-3) is the most prominent fatty acid in the brain, where it is considered functionally essential [9,10]. DHA concentration is especially high in neurons where it facilitates development and synaptic functions [11], with a special interest in human brain evolution [12]. A proper balance between omega-6 and omega-3 supplementation during pregnancy and infancy is required for correct neural development [13,14,15]. Along with aging, a decrease in long-chain PUFA levels in the brain has been observed, especially for DHA levels [16,17,18]. These deficiencies are correlated to a cognitive decline in normal aging but might be even more detrimental in pathological aging. In Alzheimer’s disease, decreases in PUFAs, particularly in essential fatty acids such as DHA, have been observed [19,20,21]. These results hint at a possible correlation between neurodegenerative diseases and cerebral DHA deficiency.

DHA has many beneficial properties, especially for cerebral diseases such as Alzheimer’s disease, that were covered in numerous reviews [22,23,24,25,26]. These include pro-neurogenic, anti-oxidative, anti-inflammatory, and anti-apoptotic properties. These potent neuroprotective effects might be partly due to the conversion of DHA into active secondary metabolites such as protectins, including protectin DX [27], resolvins, and maresins [28]. DHA can also be transformed into N-Docosahexaenoylethanolamide, an endocannabinoid-like lipid mediator that has been named synaptamide due to its capacity to induce synaptogenesis, neurogenesis, and neurite outgrowth [29,30,31]. Due to its enrichment in double bonds, DHA can also provide fluidity to cell membranes [3,32,33]. As increasing its accretion into the brain through esterification into structured phospholipids improves cognitive functions in healthy brains [34], it might also heighten its neuroprotection against neuronal death.

Since DHA biosynthesis from the essential *n*-3 precursor α-linolenic acid is very low in humans, except during pregnancy [35], the accretion of dietary DHA from blood is quite crucial. An adequate brain DHA content then depends on both food intake and blood availability [36]. Since blood DHA is present in different chemical forms, especially esterified in glycerolipids [37], its transport to the brain through the highly selective blood–brain barrier is a required step. The involvement of DHA-containing lysophosphatidylcholine (DHA-LysoPC) as an efficient transporter of DHA to the brain, as well as its metabolism and potent neuroprotective effects, is discussed in this review. This review also introduces some studies focusing on the effects of DHA-containing phospholipids on models of neurological diseases.

## 2. Transport of DHA, Esterified in Phospholipids, to the Brain

### 2.1. LysoPC as a Preferential Transporter of DHA to the Brain

Early studies have shown that plasma unsaturated LysoPC bound to plasma albumin could mainly result from liver phospholipase A_1_ activity [38,39]. It was then hypothesized that unsaturated fatty acids (UFAs) could be available to the brain from those LysoPC and non-esterified UFAs, both being bound to plasma albumin [40]. The results clearly showed that intravenously injected albumin-bound UFAs (18:1*n*-9, 18:2*n*-6 and 20:4*n*-6) were 10-fold less incorporated into rat brains than UFAs esterified at the *sn*-2 position of LysoPC [40]. Interestingly, this preferential uptake from LysoPC was not observed for the saturated fatty acid 16:0 [40]. DHA, being an abundant and crucial PUFA in the brain, has been studied in both forms (non-esterified and esterified at the *sn*-2 position of LysoPC), and its uptake by the brain was also compared with other organs. The preferential uptake of DHA esterified in LysoPC was confirmed in the brain (with at least 10-fold ratio) but not in other organs such as the heart, kidney, and liver, with an even preferential uptake of non-esterified DHA in the heart and liver [41].

The preferential brain uptake of UFAs esterified in LysoPC was later confirmed and explained through the expression of the symporter major facilitator superfamily domain-containing protein 2A (Mfsd2a), almost exclusively expressed on endothelial cells of the blood–brain barrier [42]. Mfsd2a was further studied for its 3D structure and for its interactions with choline phospholipids having a long hydrophobic chain (at least 16:0), including LysoPC, Lyso-Platelet-Activating Factor (LysoPAF), and even PAF, suggesting that the short acetyl chain in PAF does not alter the transport ability of Mfsd2a [43].

**Figure 1:**
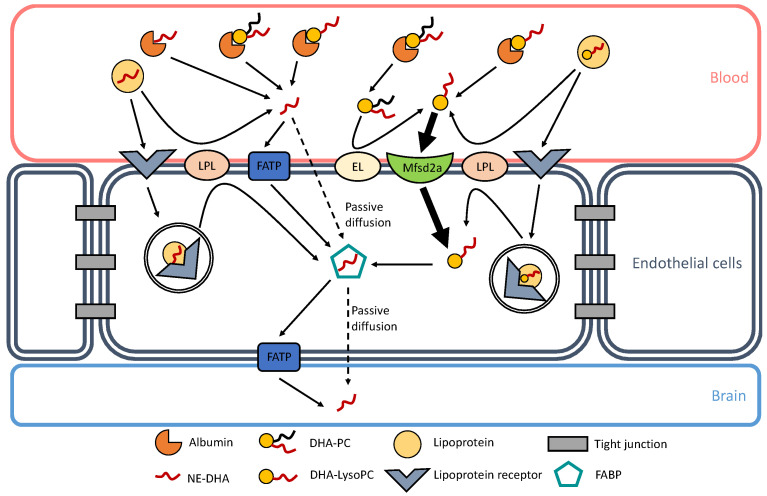
Docosahexaenoic acid (DHA) transport through the blood–brain barrier (BBB). Currently known mechanisms of DHA transport are represented in this figure. In blood, albumin can bind non-esterified DHA (NE-DHA), DHA-containing lysophosphatidylcholine (DHA-LysoPC), and DHA-containing phosphatidylcholine (DHA-PC). NE-DHA is released from albumin in the vicinity of endothelial cell membranes and is incorporated into the endothelium by passive diffusion or transportation through fatty acid transport proteins (FATP). DHA-LysoPC is also released from albumin and can be actively transported into the endothelium through the symport major facilitator superfamily domain-containing protein 2A (Mfsd2a). DHA-PC can also be released from albumin and can generate DHA-LysoP C through the action of an endothelial lipase (EL), as shown by Chen and Subbaiah [51]. Lipoproteins are other carriers of NE-DHA and DHA-LysoPC. They can release NE-DHA and DHA-LysoPC through the action of lipoprotein lipases (LPL). Lipoproteins can also bind to their receptors and go through transcystosis. Inside endothelial cells, lipoproteins can be hydrolyzed and can release either NE-DHA or DHA-LysoPC. In the endothelium, NE-DHA is bound to fatty acid binding proteins (FABP) for it to cross the intercellular space to reach brain cells. NE-DHA can either diffuse passively through the endothelial-brain barrier or be transported through FATP.

The preferential brain uptake of DHA, and other PUFAs of functional interest such as ArA, when esterified in LysoPC at the most observed physiologically *sn*-2 position is relevant because of a substantial amount of this lysolipid in plasma. Indeed, similar amounts of non-esterified ArA and DHA, and that esterified in LysoPC, are associated with rat plasma albumin [41]. This is also valid for human plasma, with about equal amounts of ArA-containing LysoPC and DHA-LysoPC associated with high-density and low-density lipoproteins as well [44]. Though higher brain accretion was observed with DHA esterified in phosphatidylcholines (PC) and phosphatidylserines (PS) compared to DHA-containing triacylglycerols [45,46], the highest brain uptake was shown with DHA-LysoPC [47]. Multiple mechanisms and factors can affect DHA esterification [48], such as hypercapnia/ischemia [49] and maternal obesity [50]. Currently known mechanisms of DHA transport through the BBB are represented in Figure 1.

In early studies comparing non-esterified PUFAs and PUFA-containing LysoPCs, the latter were *sn*-2-acyl-LysoPCs, to mimic the physiological situations in which LysoPCs are produced by the cleavage of 1-palmitoyl/stearoyl,2-arachidonoyl/docosahexaenoyl-GPC by phospholipase A_1_ or triacylglycerol lipase having a phospholipase A_1_ activity [38]. However, studies showing the involvement of Mfsd2a in LysoPC uptake did not consider the position of the acyl, or alkyl in the case of LysoPAF [43]. This suggests that the position isomers of the acyl moieties might not be crucial for the LysoPC uptake. Indeed, the incubation of *sn*-2-acyl-LysoPCs in physiological conditions leads to the migration of acyl groups from the *sn*-2 to the *sn*-1 position of LysoPCs [44,47]. This does not indicate whether one specific position isomer is required for its brain uptake, as one isomer may be converted or retro-converted into the other before being taken up. However, to maintain DHA at the supposed physiological *sn*-2 position of LysoPC, it was decided to stabilize it by esterifying the *sn*-1 position with the shortest acetyl moiety [52]. The resulting structured phospholipid, 1-acetyl,2-docosahexaenoyl-glycerophosphocholine, was named AceDoPC.

### 2.2. Stabilized Form of DHA-Containing LysoPC: AceDoPC

When ^14^C-labeled DHA in AceDoPC or ^14^C-labeled non-esterified DHA were intravenously injected into rats, there was no different ^14^C-DHA uptake by the heart and liver, but there was a significantly higher uptake of DHA in the brain from AceDoPC compared to non-esterified DHA, while both forms equally decreased from plasma [53]. Analysis of ^14^C-DHA-containing AceDoPC, PC, and phosphatidylethanolamine (PE) within the brain 1, 24, and 48 h after AceDoPC injection showed a progressively decreased AceDoPC level and increased labeled PC and PE levels [53]. Interestingly, ^14^C-LysoPC was present in small similar amounts for the three times considered, suggesting that AceDoPC was de-acetylated within the brain, and LysoPC was quickly metabolized for DHA redistribution within PC and PE [53].

An in vitro reconstituted blood–brain barrier (BBB) showed the passage of DHA esterified in AceDoPC or PC, or non-esterified DHA, across the BBB [54]. DHA crossing from AceDoPC was significantly higher than from PC, the latter being less efficient than non-esterified DHA [53]. These results are in agreement with a preferential crossing of the reconstituted BBB model by DHA-LysoPC compared to non-esterified DHA [55]. The molecular modeling of AceDoPC and DHA-LysoPC showed a very similar 3D structure [53], differing only by the acetyl moiety at the *sn*-1 position, and AceDoPC could be recognized by Mfsd2a as well, explaining similar preferential BBB crossing.

A recent study in human volunteers who ingested 50 mg of ^13^C-labeled DHA, esterified in AceDoPC or a triacylglycerol (TAG), showed that the blood bioavailability of ^13^C-DHA was higher from AceDoPC than from TAG [56]. In brief, around twice as much DHA accumulated in red cells from AceDoPC, and this preference was especially clear in PE after 6 days with a transient accumulation in PC after 3 days, which fits well with a long-term accumulation in brain PE [57] if extrapolated from red cells, which could be considered as biomarkers of DHA accumulation in the brain [58].

## 3. Neuroprotective Properties of DHA-Containing Phospholipids

### 3.1. DHA-Containing Phospholipid for the Treatment of Alzheimer’s Disease

Amyloid beta (Aβ) induced neurotoxicity can lead to the elevation of oxidative stress in the brain. In an in vitro model of Aβ1-42 neurotoxicity, primary neurons treated with PC from eggs showed less neuronal death with a reduced lactate dehydrogenase release [59]. In a rat model injected with Aβ1-40, diets enriched with DHA-containing PC (DHA-PC) or PS (DHA-PS) could increase the antioxidative enzyme superoxide dismutase (SOD) level and could reduce lipid peroxidation, inflammatory, and apoptotic levels, alongside improving spatial learning cognitive functions [60]. An increase of glutathione peroxidase (GSH-Px) and SOD activities with the improvement of cognitive deficits have also been shown in Aβ25-35-induced Alzheimer’s disease rat models treated for 30 days with DHA-PC [61]. In humans, a prospective follow-up study showed that subjects with baseline plasma DHA-PC levels in the upper quartile had 39% and 47% lower risks of developing Alzheimer disease and all-cause dementia, respectively, compared with participants with levels in the lower 3 quartiles [62].

In a study of senescence-accelerated prone 8 (SAMP8), mice were fed with a high-fat diet, as a model of Alzheimer’s disease, or with a diet enriched with DHA-PC or DHA-PS, which both increased the activity of antioxidative enzymes GSH-Px and SOD while decreasing malondialdehyde, a marker of lipid peroxidation [63]. The mice also showed enhanced cognitive performances, improved neuroprotection through decreased neuroinflammation and apoptosis, and amelioration in Aβ pathology.

The observed improvement of brain health and cognitive functions in the pathology of Alzheimer’s disease could be due not only to the neuroprotective effects of DHA (anti-inflammatory, anti-oxidative, and anti-apoptotic) but also to the beneficial transport of DHA through the BBB, increasing its bioavailability in neural cells. Another working hypothesis is that DHA can also prevent the accumulation of Aβ peptides [22,64,65,66] and the formation of fibrils [67,68,69], thus decreasing the apoptotic effects of oligomers. DHA is suggested to act on multiple pleiotropic mechanisms, leading to beneficial effects on the pathology of Alzheimer’s disease [21,23]. An additional hypothesis is that the choline moiety in the polar head of PC is crucial for neuroprotection, as Ko M. et al. reported that PC but not PS was able to protect against Aβ-induced cell toxicity [59]. It may be speculated that this choline moiety might contribute to acetylcholine production, resulting from PC hydrolysis by phospholipase D, with the resulting choline being further converted by endogenous acetyl-CoA.

### 3.2. Potential Therapy to Other Neurological Diseases

Mice treated with 1-methyl-4-phenyl-1,2,3,6- tetrahydropyridine (MPTP) to mimic oxidative damage induced by the pathology of Parkinson’s disease were fed with DHA- and eicosapentaenoic acid (EPA)-containing phospholipids, which were extracted from squid roe and contained mainly DHA-PC, DHA-containing PE (DHA-PE), and DHA-LysoPC [70]. Compared to the control group (only treated with MPTP), mice fed with DHA/EPA-PC had increased levels of antioxidative enzymes (GSH-Px and SOD) along with a reduction of motor impairments and a decrease of pro-apoptotic markers. Further study on the same model showed that a DHA-PC enriched diet could elevate activities of glutathione and SOD, alleviate the loss of dopaminergic neurons following MPTP treatment (notably through the reduction of pro-apoptotic markers), and dampen cognitive impairments in locomotor activity [71]. Parkinson’s disease is mainly characterized by the abnormal aggregation of α-synuclein protein forming Lewy bodies, an imbalance in the levels of reactive oxygen species, and the loss of dopaminergic neurons. Omega-3 fatty acids, and particularly DHA, can interact with α-synuclein to prevent its detrimental oligomerization [72,73,74]. Another pathway of action of DHA is the modulation of dopamine-induced neurodegeneration [75,76] and the enhancement of anti-oxidative pathways [76].

In a model of dementia induced by short-term memory and learning impairment by treatment with scopolamine, mice fed with squid PC (enriched in DHA) performed better in a spatial-learning memory test and had increased antioxidative activity and a lower lipid peroxidation level compared to the control group [77]. Interestingly, elevated levels of acetylcholinesterase activity induced by scopolamine injection were reduced with squid PC treatment. In a following study by the same research group, it was shown that a DHA-deficient diet could lead to further damage due to scopolamine treatment through oxidative stress, apoptosis, inflammation, and delayed neurodevelopment [78], hinting at possible preventive therapy through a balanced omega-3 diet.

The potential use of DHA’s beneficial properties on neuropsychiatric disorders is also currently under study [79]. Through its potency to reduce anti-inflammation and to promote neurogenesis, DHA was shown to reduce inflammatory markers in both in vitro and clinical studies [80]. The authors found correlations between higher levels of anti-inflammatory markers linked to DHA and lower levels of depressive symptoms. Similarly, in an in vivo study of forced swimming tests on rats, a decrease of inflammatory cytokines and an increase in serotonin levels were observed with omega-3 supplementation, suggesting anti-depressant effects of DHA [81]. Interestingly, dietary supplementation of DHA-containing phospholipids in a mice model of depression rescued depression-like behavior and inhibited neuroinflammation, suggesting increased effects on depression through DHA esterification in phospholipids [82].

### 3.3. AceDoPC as a Potential Antioxidant and Neurogenesis Inducer

In the case of AceDoPC initially, *sn2*-DHA-LysoPC was acetylated at the *sn*-1 position to prevent the migration of DHA from the *sn*-2 position as discussed above [52], but it appears that such an acetylation also confers some antioxidant activities to AceDoPC compared to non-esterified DHA. This was observed in an experimental stroke with a more significant lower size of post-stroke lesions and decreased oxidative stress after AceDoPC intravenous injection [83]. In an in vitro model of stroke on adult neural stem cells, strong antioxidant actions of AceDoPC could be seen on prostanoids and lipoxygenase product formation, with lipoxygenase products from ArA (leukotriene B4, LTB_4,_ and 15-Hydroxyeicosatetraenoic acid, 15-HETE) being surprisingly more affected than prostanoids [84].

The inhibition of prostanoid formation could be explained by the inhibition of cyclooxygenases (COX), as shown in using purified COX-1 and COX-2 [85], suggesting an aspirin-like effect of the acetyl-containing AceDoPC. Beyond these effects on lipid metabolism, the treatment of AceDoPC by phospholipase D (PLD) leads to acetylcholine formation, likely by the combination of the acetyl group of the molecule with the released choline moiety due to PLD cleavage process [85]. AceDoPC also acts as an inhibitor of lipopolysaccharide-induced neuroinflammation, both in vitro and in vivo, with some specificities compared to DHA-PC [86].

In adult neural stem cells, nanomolar concentrations of AceDoPC increased neurogenesis by 2.5 fold (compared to the control) in the presence of AceDoPC, while 1.5 fold increase with non-esterified DHA was observed [84]. Enhanced neurogenesis by AceDoPC was even higher under pathological conditions (under hypoxia/ischemia-like conditions) while no effect was observed on gliogenesis. Another phospholipid that is structurally similar to AceDoPC but contains protectin DX, a metabolite of DHA, at the *sn*-2 position, was also produced [87]. This phospholipid, labeled AceDxPC, might enhance the beneficial effects of AceDoPC.

An additional interest of AceDoPC is due to the quick loss of its acetyl moiety [53] then releasing *sn*-2-DHA- LysoPC (with DHA at the *sn*-2 position), which is quickly isomerized into *sn*-1-DHA-LysoPC (with DHA at the *sn*-1 position) [44], a substrate for producing synaptamide [88].

## 4. Conclusions

Essential omega-3 fatty acids are major constituents of cell membranes. DHA is especially prominent in neuronal cells and is necessary for the healthy neurodevelopment and healthy aging of the human brain. Since DHA shares many common signaling pathways with omega-6 ArA, the balance between omega-3 and omega-6 species can determine whether tissues will be inflamed, oxidized, or apoptotic under pathological damage. This is of particular importance in the case of neurodegenerative diseases, where proper homeostasis is required for maintaining neuron survival and cognitive functions.

PUFAs are scarcely synthesized de novo from their precursors and must be largely incorporated through a balanced diet. Since BBB is a selective barrier of the nutrients passing from blood to the brain, there is a need for new strategies to efficiently transport omega-3 PUFAs to neural cells. In summary, this review points out the role of LysoPC as a preferential transporter of DHA to the brain, particularly by crossing the BBB, likely through the Mfsd2a symporter. DHA being acylated at the *sn*-2 position of LysoPC after the cleavage of DHA-containing PC by triacylglycerol lipase/phospholipase A_1_, it mainly migrates to the *sn*-1 position with time. Preventing such a migration by acetylating the *sn-1* position led to further studies. The resulting 1-acetyl,2-docosahexaenoyl-glycerophosphocholine, AceDoPC, was then studied as a transporter of DHA to the brain. In addition to its efficiency for DHA brain uptake, AceDoPC appeared as a potential antioxidant and an acetylcholine source through its cleavage by phospholipase D.

This review also highlights the potential beneficial effects of DHA being esterified within structured phospholipids compared to its non-esterified form in the pathology of neurodegenerative diseases. Increasing the bioavailability of DHA might enhance its anti-inflammatory, anti-oxidative, and anti-apoptotic effects, but the conformation of the DHA-containing phospholipid could also exert supplementary beneficial effects on the degeneration of neuronal cells.

## Data Availability

Not applicable.
